# Corifollitropin-α compared to daily r-FSH in for patients undergoing intracytoplasmic sperm injection: Clinical trial study

**DOI:** 10.18502/ijrm.v17i1.3817

**Published:** 2019-03-07

**Authors:** Ziba Zahiri Sorouri, Davoud Pourmarzi, Niloufar Safar Khah

**Affiliations:** ^1^Reproductive Health Research Center, Department of Obstetrics & Gynecology, Al-zahra Hospital, School of Medicine, Guilan University of Medical Sciences, Rasht, Iran.; ^2^Epidemiology, Reproductive Health Research Center, Gilan University of Medical Sciences, Rasht, Iran.

**Keywords:** *Corifollitropin alfa*, * Gonal-F*, * Pregnancies*, * r-FSH*

## Abstract

**Background:**

The current treatment regimen for ovarian stimulation in Intracytoplasmic sperm injection (ICSI) patients is daily injections of Gonadotropins. Recombinant DNA technologies have produced a new recombinant molecule that is a long-acting Follicle Stimulating Hormone (FSH), named corifollitropin alfa. A single injection of long-acting FSH can replace seven daily FSH injections during the first week of controlled ovarian stimulation (COS) and can make assisted reproduction more patients-friendly. There is limited data with different results in this area.

**Objective:**

To compare the effectiveness of long-acting FSH vs. daily r-FSH in terms of pregnancy and safety outcomes in women undergoing ICSI cycles.

**Materials and Methods:**

In this clinical trial study, 109 women who were the candidates for ICSI at azzahra hospital were divided in two groups. The first group received 150 units of daily Gonal-f from second or third day of menstruation. The second group received a 150IU corifollitropin alfa on the second or third day of mensuration, and the treatment continued from day eighth of stimulation with Gonal-f based on the ultrasound finding. Both the groups received GnRH antagonist from fifth day of stimulation. Two groups were compared in terms of number of dominant follicles, number of oocytes, stimulation duration, total number of embryos, number of transferred embryos, and success rate of pregnancy.

**Results:**

No significant difference was found between the two groups in terms of stimulation duration, number of follicles, number of oocytes, total number of embryos, and number of transferred embryos. Moreover, pregnancy outcomes including chemical pregnancy rate (positive pregnancy test), clinical pregnancy rate (detection of fetal heart), the rate of ovarian hyper-stimulation syndrome, multiple-pregnancy, ectopic pregnancy, and miscarriage didn't have a significant difference between the two groups.

**Conclusion:**

As corifollitropin alfa was as effective as r-FSH, it could be used as an alternative to ovulation stimulation method in patients undergoing ICSI.

## 1. Introduction 

Assisted reproductive techniques (ART), such as in vitro fertilization (IVF)/Intracytoplasmic sperm injection (ICSI) are very useful in infertile couples in order to achieve desired fertility outcome, where ovulation stimulation is the prerequisite for using these methods. Different methods are used for ovulation stimulation, and each of them has some advantages and disadvantages (1). The most common method used in this regard is a daily injection of gonadotropins for ovulation stimulation (2). Accordingly, much effort has been made in recent years to simplify the IVF therapeutic methods in order to decrease the burden imposed on the patient and to prevent the pregnancy loss after using ART through developing a high-quality method that is patient-friendly for ovulation stimulation (3–5). Great number of researchers have recommended using corifollitropin alfa (long-acting Follicle-stimulating Hormone (FSH)) because of bioactivity profile (6). A single injection of long-acting FSH (corifollitropin alfa) could be an alternative for daily injections of FSH during the first wk. of ovulation stimulation, highly favored and welcomed by the patients (7). Long-acting impacts of this recombinant drug (corifollitropin alfa) are related to coupling of carboxy-terminal peptide of beta chain of human chorionic gonadotropin to FSH (8, 9). Other advantage of this drug is achieving the peak of serum concentration in the shortest time, speeding up its effect on ovarian stimulation (10). Accordingly, it is able to stimulate the ovulation and follicular growth by injecting a single dose of corifollitropin alfa in the first wk. of the treatment cycle. Therefore, the reduced number of injections is considered as one of its advantages (11, 12). As this drug is welcomed by patients, and given the reduced psychological stress and the number of referrals, many studies have been carried out on corifollitropin alfa by researchers in terms of its effectiveness, factors affecting the response, used dose, and people who meet the criteria to receive this drug. However, there is no theoretical agreement in this regard, and therefore further studies are required to bring out clear results (7, 6, 11, 12).

Accordingly, this study was carried out to compare the impact of corifollitropin alfa and Gonal-f in infertile patients treated by ART who were referred to an infertility center in Iran.

## 2. Materials and Methods 

### Study population 

This research is a clinical trial study that was carried out on patients who were candidate for ICSI. Women were randomly assigned to corifollitropin alfa or Gonal-f groups. The inclusion criteria of the study included age between 18–36 yr., regular menstruations (interval between 24–35 days), body mass index (BMI) between 19–30 kg/m${}^{2}$, presence of two ovaries, having an ultrasound within the last 6 wk. (so that no problem is seen in the uterus and the ovaries), hysterosalpingography or laparoscopy within last 2 yr., to check if there was a problem in the uterus (Myoma, septum, polyp), FSH on second-fourth day of menstruation below 10, normal thyroid-stimulating hormone, sperm analysis at acceptable level for ICSI (sperm count being not less than 5 million). The exclusion criteria of the study included patients who needed more than 150 units of r-FSH per day, antral follicular count more than 10–12 mm in screening ultrasound in second or third days of menstruation, inappropriate response to gonadotropins, and inappropriate number of retrieved oocyte (less than 3 oocytes) in previous ART cycles, 3 or more than 3 previous failed ART, lack of fertilization in previous ICSI, previous ICSI history with inadequate sperm number, leading to taking sperm from the testicles and epididymis, history of endometriosis stage III or IV, presence of unilateral or bilateral hydrosalpinx, presence of any underlying disease (liver, and kidney), and smoking. Among 315 patients who were candidate for ICSI 109 patients were eligible for study.

### Ovarian stimulation

Transvaginal ultrasound was used for all participants on the second or third day of menstruation (and in those whose ultrasound was normal -normal uterus and ovaries without cyst). Patients were randomly divided in two groups. The first group received 150 units of daily Gonal-f from second or third day of menstruation by using the conventional method of ovulation in the antagonist protocol. According to the fixed protocol of antagonist, Gonadotropin-releasing hormone (GnRh) antagonist was injected subcutaneously on a daily basis from the fifth day of starting the Gonal-f (Ganirelix 0.25 mg in 0.5 cc daily). Then, serial ultrasounds were performed based on the ovarian results. In the second group, a subcutaneous dose (150 IU) of corifollitropin alfa (USA/MSD/Elnova) was injected in the second or third day of menstruation, and GnRh antagonist (Orgalutran, Merk, USA) was injected subcutaneously on daily basis, as it was performed in the first group on the fifth day. The treatment continued from day eighth of stimulation with Gonal-F and the dose based on ultrasound findings. Then, 250 µg of Ovitrelle (Merck-Serono, Italy) was injected to both groups by observing at least three follicles at size of 17 mm and larger. After 36 to 38 hr, ovarian puncture was performed under ultrasound guide, and the taken oocytes were prepared for ICSI. In each of the two groups, embryo were transferred to the uterus, 2–3 days after ovum pick up (OPU), and not more than three embryo were transferred. All patients underwent luteal phase support by injecting 100 mg per day progesterone (progesterone, Iran hormone, Iran) from the day of OPU and 150 mg per day after embryo transfer. The exclusion criteria of the study included less than three dominant follicles, lack of transfer of embryos due to high progesterone or ovarian hyper-stimulation syndrome.

### Ultrasound assessment of follicular development

As mentioned before, the 1${}^{st}$ ultrasound was done on the 2${}^{nd}$ or 3${}^{rd}$ day of cycle and after that in 5${}^{th}$ day of stimulation. Then, serial ultrasounds were done based on patient's response to ovarian stimulation.

### Outcome measures 

The primary outcome measure was the rate of clinical pregnancy. The secondary outcome measures included the duration of stimulation, number of dominant follicles, number of oocytes, total number of embryos, number of transferred embryos and the rate of positive β-HCG, miscarriage, multiple-pregnancy, and Ovarian Hyper-stimulation Syndrome (OHSS).

### Sample size 

The sample size was calculated based on the reported fertility success rates of 41% with recombinant FSH (150 IU/d) and 16% with corifollitropin alfa (120 μg) (13), 10% drop-out rate, 80% power, and a significance level of 5%, which indicated that 54 participants in each group were needed.

### Ethical consideration

After approving the project by Ethics Committee of the University of Medical Sciences, IR and registering it in the Iranian Clinical Trial Database, IR, the sampling process was initiated. The methodology of the study was explained to all participants and the consent to participate in the study was obtained through a consent form filled by each one of them.

### Statistical analysis

Data were analyzed using SPSS software (Statistical Package for the Social Sciences, version 21.0, SPSS Inc, Chicago, Illinois, USA) and descriptive and analytical statistics. Independent *t*-test was used to compare the mean of quantitative data between the two groups. In addition, Fisher and Chi-square exact tests were used in order to compare qualitative and classified data between the two groups. The statistical significances were considered as p < 0.05.

**Figure 1:**
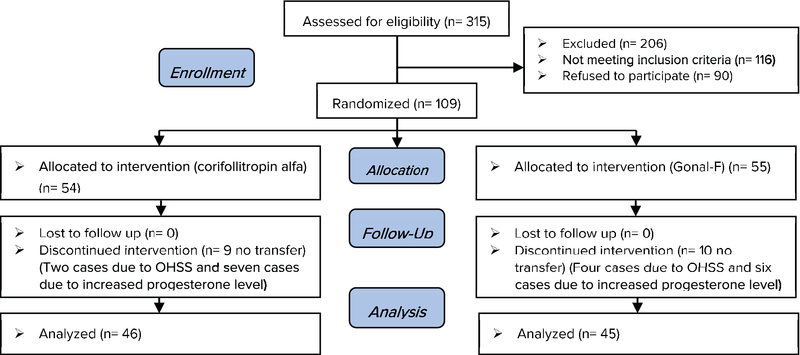
Study by Consort flowchart.

In this study, 109 women participated, of which, 55 were assigned to Gonal-F group and 54 to corifollitropin alfa group (Figure 1).

## 3. Results 

In this study, the two groups had similar mean age, BMI, and also they were similar in terms of infertility duration, infertility type, and infertility cause. The main characteristics of the patients in two groups are illustrated in Table I. No difference was found between the two groups in terms of stimulation duration, number of follicles, number of oocytes, total number of embryos, and number of transferred embryos Table II. No significant difference was found between the two groups in terms of chemical and clinical pregnancy, miscarriage under 14 weeks, OHSS, and multiple-pregnancy (Table III).

**Table 1:** Demographic characteristics of two groups who were treated with corifollitropin alfa and Gonal-F.


**Variable**		**Group receiving Gonal-F**	**Group receiving long-acting FSH**	**p-value**
Body Mass Index${}^{a}$		2.55 ± 25.06	2.56 ± 25.48	0.389*
Age (yr)${}^{a}$		4.51± 31.36	4.58 ± 31.18	0.838*
Infertility duration (yr)${}^{a}$		3.79 ± 4.45	3.4 ± 4.61	0.582**
Infertility type${}^{b}$			0.161***
Primary		87.3 (48)	96.3 (52)	
Secondary		12.7 (7)	3.7 (2)	
Infertility cause${}^{b}$			0.957***
	Male	29.1 (16)	33.3 (18)	
	Female	36.4 (20)	35.2 (19)	
	Female and male	21.8 (12)	18.5 (10)	
	Unknown	12.7 (7)	13 (7)	
Note: ${}^{a}$Data presented as mean ± SD; ${}^{b}$Data presented as *n* (%); *t-test; **Mann-Whitney U test; ***Fisher's exact test;				
FSH: Follicle-stimulating Hormone.				

**Table 2:** Comparison of variables related to fertility in the two groups who were treated with corifollitropin alfa and Gonal-F.


**Variable**		**Mean ± SD**	**p-value**
Duration of ovulation stimulation (day)			
	Receiving Gonal-F	10 ± 2.3	
	Receiving long-acting FSH	9.77 ± 1.3	0.802
Number of follicles			
	Receiving Gonal-F	15.2 ± 7.05	
	Receiving long-acting FSH	12.55 ± 4.53	0.351
Number of oocyte			
	Receiving Gonal-F	11.1 ± 4.7	
	Receiving long-acting FSH	10.66 ± 6.85	0.873
Total number of embryos			
	Receiving Gonal-F	6.9 ± 3.31	
	Receiving long-acting FSH	4.77 ± 2.61	0.134	
Number of transferred embryos			
	Receiving Gonal-F	2.61 ± 0.78	
	Receiving long-acting FSH	2.64 ± 0.58	0.814	
Number of Gonal-F injections			
	Receiving Gonal-F	28.6 ± 5.48	
	Receiving long-acting FSH	11.11 ± 3.17	0.0001	
Note: Data presented as mean; *T-Test;			
FSH: Follicle-stimulating hormone			

**Table 3:** Frequency distribution of pregnancy consequences in two groups treated with corifollitropin alfa and Gonal-F.


**Variable**		**Receiving Gonal-F**	**Receiving long-acting FSH**	**p-value**
Chemical pregnancy				
	Yes	18 (32.7)	18 (33.3)	
	No	37 (67.3)	36 (66.7)	0.946*	
Clinical pregnancy				
	Yes	13 (23.6)	15 (27.8)	
	No	42 (76.4)	39 (72.2)	0.621**
OHSS				
	Yes	4 (7.3)	2 (3.7)	
	No	51 (92.7)	52 (96.3)	0.67*
Increased progesterone levels				
	Yes	6 (10.9)	7 (13)	
	No	49 (89.1)	47 (87)	0.741*
Miscarriage				
	Yes	4 (7.3)	3 (5.6)	
	No	51 (92.7)	51 (94.4)	0.715**
multiple-pregnancy				
	yes	2 (3.6)	5 (9.3)	
	no	53 (96.4)	49 (90.7)	0.271**
Note: Data presented as *n *(%); *Chi Square; **Fisher's exact test;				
FSH: Follicle-stimulating Hormone;				
OHSS: Ovarian Hyper-stimulation Syndrome.				

## 4. Discussion

Given the increasing developments in ART to simplify the course of patients' treatments and their frequent referrals, much effort has been made by researchers to decrease the psychological stress imposed on patient in order to improve the response to treatment. In some of the studies conducted in recent years, corifollitropin alfa has been recommended as a good alternative because of less number of injections and reduced psychological burden and less follow-up, in comparison to the daily FSH method. However, there is still no consensus on efficiency, used dose, and the factors affecting the response and selection of people meeting the inclusion criteria and the standard protocol (6, 7, 11, 12, 14). Thus, this study was carried out to evaluate the effectiveness of long-acting FSH (corifollitropin alfa) versus daily injection of Gonal-f in patients undergoing ICSI.

Findings revealed that all pregnancy outcomes and other variables such as ovulation stimulation duration, number of follicles, number of oocytes, total number of embryos, and the number of transferred embryos, chemical and clinical pregnancy, miscarriage under 14 wk. and outcomes such as ovarian hyper-stimulation syndrome, increased progesterone level above 1.5–2 ng/ml and multiple-pregnancy were similar between the two groups, and no superiority was found over the previous protocol. This finding is in line with the findings of several studies (6, 7, 14–16). However, each of these studies had differences with the present study, and the findings of these studies cannot be compared decisively. In the study conducted by Pouwer, Farquhar and Kremer (7), they reported that the rate of live birth, ongoing pregnancy-OHSS, miscarriage and ectopic pregnancy did not differ in the two groups at a dose of 150–200 μg. However, in a lower dose (120–160 μg), the success rate of live birth was lower compared to the previous method. But this study was carried out as a review study with unexplained infertility cause. In another study conducted by Kolibianakis (15), he stated that there is no difference between two groups in terms of ovum taken from the ovary and the number of live birth. This study differed from the present study in terms of the participating patients, since it examined poor responder patients. Finally, this study recommended that long-acting FSH can be used to simplify ovulation stimulation in IVF, which can decrease the burden of treatment on poor responders. In another study, it was reported that the ongoing rate was similar and it was slightly higher in the long-acting FSH method compared to the daily FSH injection, and the number of oocytes taken in the long-acting FSH method was slightly more than that in the daily conventional method (16).

Selman and Rinaldi recommended that ovarian stimulation using long-acting FSH method seems to be as effective as the daily injection of FSH in patients with poor response, since no significant difference was found between the two treatment groups in terms of the number of retrieved oocytes, the number of embryos of the cleavage stage, and the rate of pregnancy and abortion (6). In another similar study, findings revealed that there is no difference between the two groups in terms of mean age (34 years) and ovulation stimulation duration (11 days), the number of oocytes, and the oocyte of metaphase 2 stage, fertility rate, chemical pregnancy, and the rate of embryo replacement. However, 100 μg long-acting FSH was prescribed for patients under 60 kg, while 150 μg of long-acting FSH was prescribed for those who were above 60 kg, and the number of subjects were not same in the two groups (26 subjects in the long-acting group versus 106 subjects in the daily Gonal-f injection group), making it difficult to compare findings of the two studies (13).

On the other hand, other studies have been carried out whose findings are different from the findings of the present study, and it has been stated that long-acting FSH has been effective in ovulation stimulation and fertility outcomes in comparison to the Gonal-F method. However, the design of majority of these studies was different from that of the present study in terms of population size and subjects participating in the study (17–20). In the meta-analysis conducted by Fensore, it was found that most of the clinical parameters, including live birth, ongoing pregnancy, and clinical pregnancy, were similar between the two groups. However, some variables such as the number of oocytes in metaphase II and embryo formation were higher in the women receiving corifollitropin alfa, and the cancellation was higher due to hyperstimulation. However, this study was conducted with a larger sample size and among the subjects who underwent IVF with donated ovum and poor response to treatment; Fensore reported that the impact of specific groups with the potentiality of hyper response should be used with caution given an increase in OHSS in the long-acting FSH group (18).

Barroso-Villa also reported the rate of successful pregnancy higher in the long-acting FSH group, which might be due to the type of treatment group selected, since they were selected among the patients who failed in previous treatment protocol (19). Benchabane and colleagues also reported that long-acting FSH had similar effectiveness with daily FSH in donated oocytes (17). In another meta-analysis, evidence showed no difference between long-acting FSH and Gonal-f in terms of ongoing pregnancy. However, OHSS was higher in the FSH group. They reported that long-acting FSH could be used as an alternative to daily FSH in patients with normal ovarian responses in IVF or ICSI, due to having the same level of effectiveness with daily FSH (18). Among the studies carried out in this regard, only the study conducted by Devroey was similar to the present study in terms of the conditions in which studies were performed, while its findings were somewhat different. Devroey and co-workers did not find any difference in ongoing pregnancy and OHSS, ovulation stimulation duration, but the risk of multiple-pregnancy in the corifollitropin group was slightly higher in the study, which was justified by more implantation of embryo (21).

In the current study, the number of multiple-pregnancy was slightly higher, but it was not statistically significant. However, further studies with larger sample size should be carried out in order to make conclusion more decisively in this regard. In another study, completely contradictory findings with the present study and previous studies were reported, which questioned the effectiveness of long-acting FSH. It suggests that long-acting FSH does not have the required effectiveness for ovulation stimulation and it cannot be a good alternative to Gonal-F. Siristatids found that live birth rate in the long-acting FSH group was lower than that in the previous protocol, but other parameters such as clinical pregnancy, abortion, and secondary complications were same in the two groups (22).

Given the different studies and obtaining contradictory results, a number of studies examined the factors involved in the response to the treatment to justify these differences. Some of these factors predicting the response to treatment effectively in long-acting FSH are the blood level of the anti-Mullerian hormone, the number of antral follicles, and the age and length of the menstrual cycle, which can weaken or exacerbate response to treatment (23, 24). As different communities were studied in various studies and different results were obtained, one cannot make decisive conclusion in this regard. However, based on the majority of studies conducted in this regard, long-acting FSH can be introduced as an appropriate alternative to Gonal-f if those individuals are selected who meet the inclusion criteria. However, it is recommended that more studies be carried out with higher sample sizes to investigate the effectiveness of the factors affecting it.

## 5. Conclusion 

Considering the fact that frequent injections of daily Gonal-F increase the risk of incorrect injection and it can affect the IVF outcome, using a drug with fewer injections and a single dose can resolve this problem. On the other hand, frequent injections increase the patients' stress and it can affect the treatment process and IVF outcome. Thus, corifollitropin alfa can be a good alternative for frequent injections. However, a study with wider dimensions and with larger sample size should be performed for better analysis and investigation.

##  Conflict of Interest

The authors declare that they have no conflicts of interest.
